# Cardiac autonomic modulation in response to postural transition during a virtual reality task in individuals with spinal cord injury: A cross-sectional study

**DOI:** 10.1371/journal.pone.0283820

**Published:** 2023-04-13

**Authors:** Denise Cardoso Ribeiro Papa, Lilian Del Ciello de Menezes, Íbis Ariana Peña de Moraes, Ana Clara Silveira, Natalia Padula, Suellen de Oliveira Veronez Silva, Roberta Caveiro Gaspar, Eduardo Dati Dias, Celso Ferreira, Luciano Vieira de Araújo, Todd A. Astorino, Helen Dawes, Carlos Bandeira de Mello Monteiro, Talita Dias da Silva

**Affiliations:** 1 Postgraduate Program in Medicine (Cardiology) at Escola Paulista de Medicina, Federal University of São Paulo (EPM / UNIFESP), São Paulo, São Paulo, Brazil; 2 Faculty of Medicine, City of São Paulo University (UNICID), São Paulo, São Paulo, Brazil; 3 Postgraduate Program in Rehabilitation Sciences, Faculty of Medicine, University of São Paulo (FMUSP), São Paulo, São Paulo, Brazil; 4 Exeter Biomedical Research Centre, College of Medicine and Health, University of Exeter, Exeter, United Kingdom; 5 Postgraduate Program in Physical Activity Sciences, School of Arts, Science and Humanities of University of São Paulo (EACH-USP), São Paulo, São Paulo, Brazil; 6 Acreditando - Center for Neuromotor Recovery, Health, and Wellness, Brazil; 7 Postgraduate Program in Information Systems, School of Arts, Science and Humanities of University of São Paulo (EACH-USP), São Paulo, São Paulo, Brazil; 8 Department of Kinesiology, California State University San Marcos (CSUSM), San Marcos, California, United States of America; 9 Department of Paediatrics, University of Oxford, Oxford, United Kingdom; University of Catanzaro: Universita degli Studi Magna Graecia di Catanzaro, ITALY

## Abstract

**Purpose:**

The postural transition from sitting to standing is a moment of dysautonomic occurrence in individuals with Spinal Cord Injury (SCI). Different tools can be used to minimize this event, such as virtual reality. Thus, we aimed to analyze cardiac autonomic modulation in individuals with SCI during postural transition from the sitting to orthostatism position using a cognitive virtual reality (VR) task.

**Methods:**

Individuals with and without SCI were positioned on the Easy Stand^®^ device, sitting at rest, at 0° considering the angle between the seat and the floor, elevation at 45°, and orthostatism at 90°, for 5 minutes in each position. Heart rate variability (HRV) measures of sympathovagal balance were collected (heart rate receiver: Polar V800). The groups were subdivided into two groups, one that performed VR as an intervention during the postural angle changes and another group that did not perform VR.

**Results:**

We evaluated 76 individuals, 40 with a medical diagnosis of SCI and 36 who composed the able-bodied control group without SCI, matched by age and sex. The HRV results showed that the SCI group who performed the task in VR demonstrated no significant difference in parasympathetic activation and global variability between the sitting versus 90° positions. There was better sympathovagal balance in SCI and able-bodied control groups who performed the VR task between the sitting versus 90° positions.

**Conclusion:**

The use of a VR task seems to contribute to better sympathovagal balance, with the potential to reduce dysautonomia during postural changes.

## Introduction

Spinal cord injury (SCI) is an incapacitating injury that compromises the structures contained in the spinal canal and can lead to alterations in motor, sensorial, and autonomic functions [1]. Although estimates of SCI prevalence range widely, the incidence rate was between 52 and 54 cases per 1,000,000 based on National Inpatient Service data. In addition, there is a high range of direct hospitalization costs per year due to the high potential of SCI to cause disability [2].

SCIs encompass a constellation of clinical conditions characterized by damage to the spinal cord, with major determinants of disability. According to Invernizzi et al. [3], the most frequent undesirable outcomes in these patients are an increased risk of fracture, disability, socioeconomic frailty, and reduced quality of life.

Another important consequence resulting from SCI is damage to the autonomic pathways that travel through the spinal column, altering the regulation of cardiovascular functions, in addition to causing changes in peripheral vasomotor responses and systemic blood flow control [4]. These cardiovascular disorders are the main causes of morbidity and mortality in acute and chronic stages of SCI [5]. The complications of cardiovascular dysfunction, following the immediate response to injury, are responsible for decreased sympathetic activity and cardiac output, as well as total peripheral blood vessel resistance, with chronic effects such as reduced resting arterial pressure, orthostatic hypotension, cardiac dysrhythmias, and autonomic dysreflexia [6].

To quantify the autonomic alterations of the cardiovascular system, heart rate variability (HRV) is a practical and effective non-invasive measurement, which describes the oscillations in the intervals between consecutive heartbeats [7–11]. Changes in HRV patterns are widely correlated with increased cardiovascular risk and sudden death [12–15].

It is known that individuals with SCI present reduced HRV and decreased resting heart rate, however, the parasympathetic and sympathetic nervous system profiles need to be more deeply explored [12, 16–19]. Studies of HRV in SCI are limited and few describe the behavior of the autonomic nervous system (ANS) in postural changes, especially in relation to orthostatism [20–22]. Despite the problems seen in clinical practice that postural changes can bring in the daily life of patients with SCI due to impacts on peripheral and cerebral oxygenation, there are no reports in the literature of non-invasive treatments that can reduce the impact of dysautonomia during postural changes.

Multiple promising therapies are actively being explored as treatments, including pharmacological, neuroprotection, and neuroregeneration, as well as neurorehabilitation, which includes Functional Electrical Stimulation, Epidural Spinal Cord Stimulation, Exoskeleton, Robotics, and traditional rehabilitation strategies including range of motion and strengthening exercises, bed mobility, transfer exercises along with locomotor training, body-weight supported treadmill training, and braces and orthoses [23–25]. Despite these possibilities of treatment, high costs, and reduced motivation, and engagement during the rehabilitation intervention are still a problem and new technologies such as Virtual Reality (VR) may be effective tools used in individuals with SCI [26].

The use of VR in rehabilitation is a modern treatment concept, based on the use of games and tasks in virtual environments, that stimulate physical or cognitive functions in individuals with different types of disabilities [27–31]. The advantages of VR are numerous and include the possibility of the activity being adapted to the individual and associated with greater motivation and engagement of patients in rehabilitation programs [32–34]. A systematic review showed a positive impact of exergames and VR on cognitive impairment patients. Thus, these innovative and technological interventions might form part of the complex and integrative rehabilitation programs developed for individuals with SCI [35].

Regarding the influence of virtual reality in rehabilitation, Alvarez et al. [27] observed that cognitively demanding computational virtual tasks are impacted by cardiac autonomic responses in individuals with disabilities who have reduced HRV and resting autonomic nervous system (ANS) adaptive capacity. Dias et al. [26], analyzed individuals with SCI (athletes and non- athletes) and found that a task using virtual reality provided adequate autonomic nervous system (ANS) adaptation during rest and physical activity only for the athletes (i.e., the study indicates the importance of practicing physical activity for individuals with SCI and a VR task is an interesting possibility).

Considering the importance of posture and physical activity for individuals with SCI we designed this study to analyze HRV during a VR task with the objective of: (1) characterizing the autonomic modulation of individuals with SCI during postural change from sitting (rest—0% on the easy stand device) to orthostatism (90° on the easy stand device) and at the elevation angle between these two positions (45° on the easy stand device); (2) comparing HRV indices at rest and while performing a motor-cognitive virtual task; (3) comparing HRV indices between SCI and able-bodied individuals.

We hypothesized that postural changes influence the HRV in individuals with SCI and that the use of a motor-cognitive virtual task will provide better autonomic adaptation. In addition, we hypothesized that the SCI individuals would present lower HRV compared with the able-bodied individuals in all postures.

## Methods

The research project was approved by the Research Ethics Committee of the Federal University of São Paulo (CAAE: 83221818.2.0000.5505) and registered at Registro Brasileiro de Ensaios Clínicos (ReBEC) database number identifier: RBR-2gzgys. Informed consent was obtained from all subjects involved in the study and the collections were performed after the research participant signed the Informed Consent Form. A cross-sectional, two group (able-bodied control and SCI), quasi-experimental study was carried out. in which groups were split into subgroups receiving or not a VR intervention. Recruitment of the experimental group was carried out at the Acreditando—Center for Neuromotor Recovery, Health and Wellness, and the protocol was applied before the rehabilitation activities. The study intervention period was between January 2019 and February 2020.

### Inclusivity in global research

Additional information regarding the ethical, cultural, and scientific considerations specific to inclusivity in global research is included in the Supporting Information (S1 Checklist).

### Participants

Individuals with a medical diagnosis of spinal cord injury, who agreed to participate in the study were considered eligible. To compose the able-bodied control group, individuals were matched by sex and age with the experimental group.

Individuals with previous heart disease and/or who used prescribed drugs that interfere with the ANS, such as antiarrhythmic drugs, as well as conditions that contraindicate the orthostatic position (myotendinous shortening and joint deformities) were not included. Failure to complete the protocol resulted in exclusion of the participant.

### Sample characterization

Demographic data were collected from the medical records and information provided by the participant. For the classification of impairment based on the completeness of the lesion and motor-sensory function, the ASIA scale (American Spinal Injury Association Impairment Scale) was applied [36, 37] and to evaluate functioning the Functional independence measure—FIM scale [38, 39] and the Spinal cord independence measure—SCIM III were applied [40, 41]. After the initial assessment, the chest strap was attached to the participants’ chest and the heart rate receiver (V800, Polar) to their wrists for the entire assessment; furthermore, the participants were instructed to abstain from beverages containing caffeine or alcohol for 24 hours before the measurements [14].

### Virtual cognitive task—Intervention

**Fig 1:**
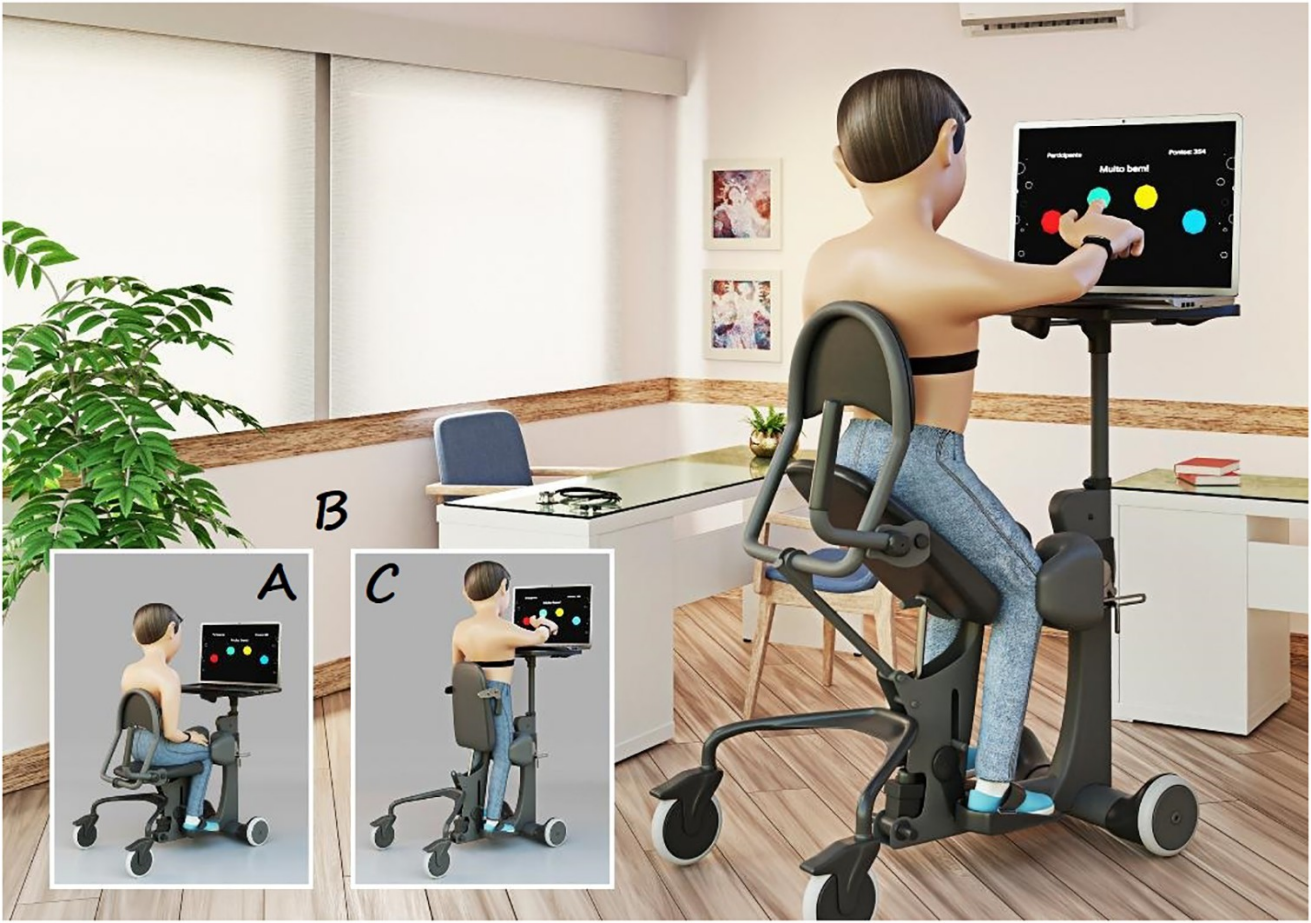
Illustrative drawing of the Easy Stand^®^. Representative design of the accomplishment of the Virtual Cognitive Task using the Touch Screen interface, wearing the Polar V800 chest strap heart rate receiver device. [A] sitting at rest at 0°, [B] in elevation at 45° while performing the task, [C] orthostatism at 90° while performing the task. All degrees refer to the angle between the seat and the floor.

The VR intervention consists of a game that analyzes the cognitive performance of the participants through a memory activity, provided by Genius software, developed by the PATER group (research and technological application group), which uses a sequence of colors emitted by the computer. The task consists of four circles (red, green, yellow, and blue) that randomly light up. The individual is required to memorize the sequence and then repeat it with the goal of reproducing the correct sequence generated. Each participant was instructed to touch the computer screen with the dominant upper limb (while the other limb remained supported on the "easy stand" device) and perform the task for 5 minutes in the elevated position (45°) and an additional 5 minutes during orthostatism (90°), at an appropriate speed (Fig 1).

### Study design

**Fig 2:**
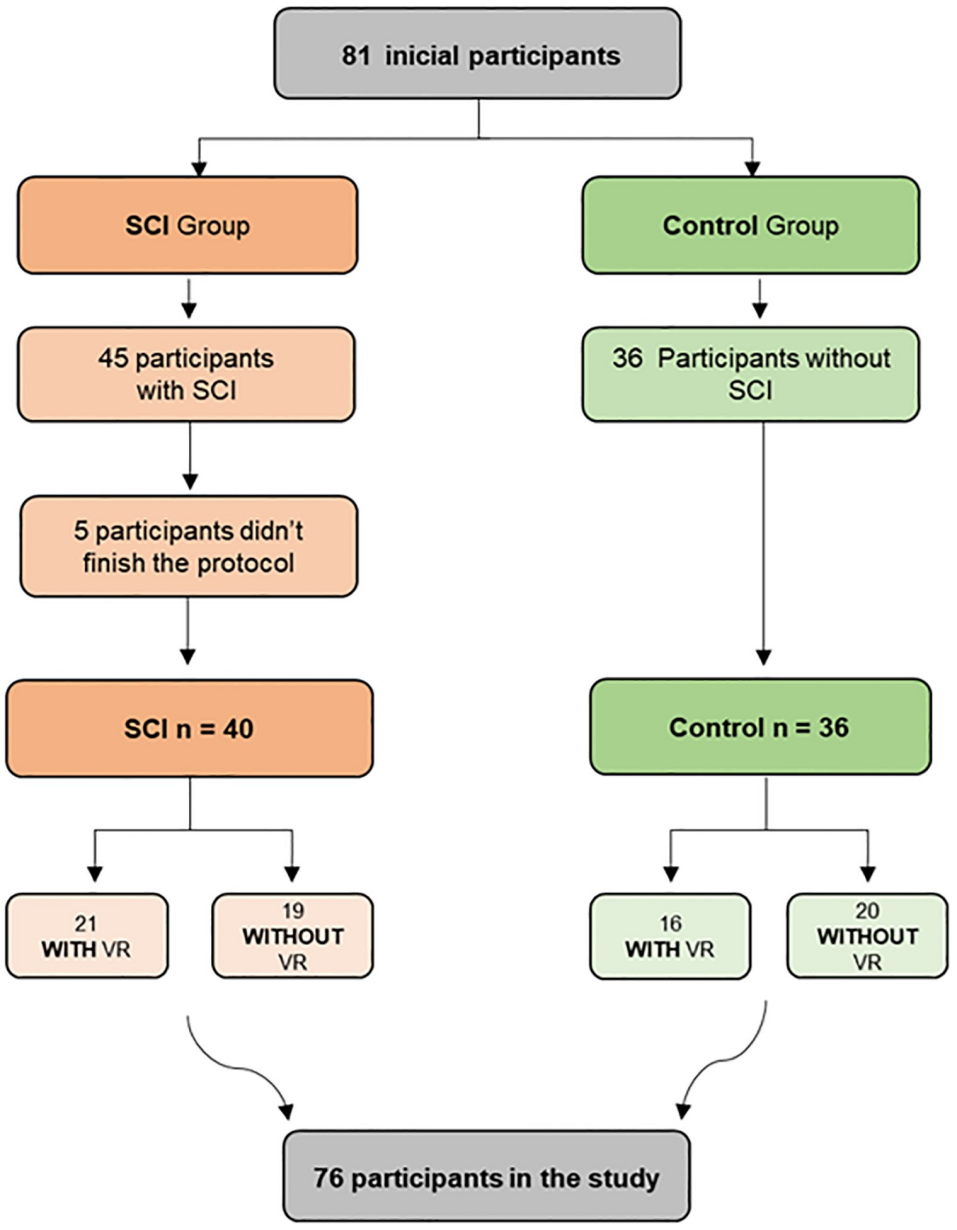
Flowchart. SCI: Spinal Cord Injury; VR: Virtual Reality.

The individuals were positioned seated on the Easy Stand^®^ device, a mobile unit that allows gradual vertical elevation from a sitting position to a standing position, with the desired angles marked and secure adjustments made to the feet, knees, and lifting belt (Fig 2). Initially, the individuals remained sitting at rest (0° considering the angle between the seat and the floor) for 5 minutes, after which they were elevated at 45° for 5 minutes, and finally orthostatism (90°) for another 5 minutes. During the five minutes at the final angles of 45° and 90°, the SCI and able-bodied control groups were subdivided randomly into two groups, one used VR as an intervention through a motor-cognitive task and the other did not perform the task (able-bodied control with and without VR, and SCI with and without VR). We randomized the groups using the website “randomization.com”, then inserted the randomization sequence into a table and followed this sequence according to the recruitment of each patient.

### Heart rate variability—Outcomes

For analysis of the HRV data, 256 consecutive RR intervals (beat-to-beat intervals) were used at rest (sitting) and during the postural changes. Manual filtering was performed using the Microsoft Excel program to eliminate premature ectopic beats and artifacts; there was no substitution, only the elimination of artifacts, and only series with 95% sinus beats were included [42]. The software used to evaluate HRV was Kubios HRV. The analysis of heart rate variability was performed by means of linear methods, analyzed in the time and frequency domains, and by nonlinear methods.

In the time domain, the indices Mean RR: mean of the normal RR intervals, Mean HR: mean of the HR, RMSSD: square of the root mean difference between adjacent normal RR intervals, which reflects the parasympathetic branch, pNN50: percentage of successive R-R interval differences whose absolute value exceeds 50ms, which reflects the parasympathetic branch, and SDNN: standard deviation of the mean of all normal RR intervals, reflecting the participation of both ANS branches [43–45].

To analyze the HRV in the frequency domain we used the spectral components of low frequency, LF—ranging from 0.04 to 0.15 Hertz, which reflects both branches of the ANS, with predominance of the sympathetic, and high frequency HF—ranging from 0.15 to 0.4 Hertz, which reflects only the parasympathetic, in absolute and normalized units, and the ratio between these components (LF/HF), which represents the relative value of each spectral component in relation to the total power. The Fast Fourier Transform (256 s window with 50% overlap) algorithm was used for the spectral analysis [8, 43].

To analyze HRV using a non-linear method, we used the Poincaré plot (components SD1, SD2, and SD2/SD1 ratio), which is a quantitative method of analysis, based on changes in sympathetic or parasympathetic modulation of heart rate on subsequent intervals, without requiring the property of stationarity of the data [46]. The SD1 index refers to the short-term standard deviation of instantaneous beat-to-beat variability, and is thus a measure of rapid changes in R-R intervals and with effects on the parasympathetic nervous system. SD2 is the long-term standard deviation of continuous R-R intervals, influenced by parasympathetic and sympathetic branches. The ratio between them, SD2/SD1, is the ratio of the short-term range to the long-term range [47, 48].

The sympathetic index (SNSi) and parasympathetic index (PNSi) have been proposed to separately assess the synergistic functions of the autonomic nervous system, using the timing of the heartbeat from the observation that cholinergic and adrenergic impulses have different temporal dynamics. The PNSi is calculated based on three parameters: 1. mean RR interval; 2. RMSSD, with high values of this index indicating high parasympathetic cardiac activation; 3. Poincaré plot index SD1 in normalized units. The SNSi index, on the other hand, is calculated based on: 1. mean HR range (higher heart rate is associated with higher sympathetic cardiac activation); 2. Baevsky stress index (Stressi), a geometric measure of HRV that reflects the stress of the cardiovascular system [49], with high Stressi values indicating reduced variability and high sympathetic cardiac activation; 3. Poincaré SD2 plot index in normalized units [50–52].

Values of PNSi and SNSi around zero indicate that the parameters reflecting the respective activity (parasympathetic and sympathetic) are, on average, equal to the normal population mean. Correspondingly, a positive index value reflects parameters above the normal population mean and a negative value, parameters below the normal population mean. During stress or high-intensity exercise, the SNS index can have values as high as 5 [49–50]. The software used for the analysis was Kubios HRV.

### Statistical analysis

Descriptive statistics were performed. Categorical data are expressed as absolute and relative frequencies, and continuous data as mean and standard error in the graphs and as mean, standard deviation (SD), and confidence interval (CI) in the Supporting Information (S1 Table). For analysis of HRV indices, Multivariate analysis of variance (MANOVA) was used for analysis between Groups (SCI and able-bodied controls), Virtual Reality (with and without), and with repeated measures for Moments (Sitting rest 0°, elevation 45°, and orthostatism 90°). We used Pearson’s Correlation to test the strength of the associations between HRV indices (RMSSD, SDNN, and SD1) and age, Neurological Level (continuous), ASIA, SCIM-III, and FIM. The partial eta-squared (ŋ_p_^2^) measures the effect size and was interpreted as small (effect size> 0.01), medium (effect size> 0.06), or large (effect size> 0.14) [53]. The observed power was also reported (OP). Values of p <0.05 were considered significant. The statistical package used was Statistical Package for Social Sciences (SPSS; IBM, Chicago, Illinois, USA), version 26.0.

## Results

A total of 83 potential participants were assessed, of which two did not meet the inclusion criteria, leaving 81 who were invited to participate in this study. However, five did not finish the protocol and were excluded from the sample. Finally, 76 patients were included in the data analysis, divided into two groups (SCI group and able-bodied control group) and two subgroups (with VR and without VR): 40 individuals in the experimental group with a medical diagnosis of SCI and 36 individuals without SCI, to compose the able-bodied control group, matched by age and sex with the experimental group (Fig 2).

The characterization of the sample is presented in Table 1. When comparing the two groups (four subgroups), there were no significant differences regarding age and sex, and the neurological level was similar when considering continuous data.

**Table 1:** Characterization of the sample.

	SCI without VR	SCI with VR	Able-bodied control without VR	Able-bodied control with VR	p-value
	(n = 19)	(n = 21)	(n = 20)	(n = 16)	
	Mean (SD)	Mean (SD)	Mean (SD)	Mean (SD)	
	[CI]	[CI]	[CI]	[CI]	
**Age (years)**	43.8 (15.3)	33.3 (11.0)	42.5 (14.0)	31.8 (10.9)	0.778
	[36.2–51.2]	[29.1–37.9]	[36.8–48.8]	[26.8–37.1]	
**Neurological level**	9 (7)	14 (5)	-	-	0.112
	6–14]	[11–16]			
**FIM**	76.8 (32.7)	92.8 (26.9)	-	-	0.167
	[58.1–93.7]	[79.8–105.2]			
**SCIM-II**	49.7 (20.4)	54.5 (22.5)	-	-	0.635
	[38.9–61.8]	[43.0–65.2]			
**Sex**	n (%)		n (%)		
Male	13 (68.4)	12 (57.1)	13 (65)	9 (56,2)	0.545
Female	6 (31.6)	9 (42.9)	7 (35)	7 (43,8)	
**ASIA**					
A	14 (73.7%)	8 (38.0)			0.019
B	0 (0)	2 (9.6)			
C	2 (10.5%)	10 (47.6)			
D	3 (15.8%)	1 (4.8)			

SCI (Spinal Cord Injury) F; Data presented as Mean (Standard Deviation—SD) and [95% confidence interval—CI], followed by One-Way ANOVA p-value for age with Minimum Significant Difference (MSD) as post-hoc test; independent t-test for Neurological level, Functional Independence Measurement Scale (FIM), and Spinal Cord Independence Measure (SCIM III) (between SCI groups); chi-square with Bonferroni as post-hoc test (for comparison between groups) for sex, and the American Spinal Injury Association impairment Scale (ASIA).

### Heart rate variability

The MANOVA found a significant effect for Moments (Wilk’s Lambda: 0.108, F_32, 38_ = 2.42, p = 0.005, ŋ_p_^2^ = 0.67, OP = 0.99), a marginally significant interaction between Moments and Group (Wilk’s Lambda: 0.173, F_32, 38_ = 1.67, p = 0.066, ŋ_p_^2^ = 0.58, OP = 0.92), and a significant interaction between Moments, Group, and VR (Wilk’s Lambda: 0.110, F_32, 38_ = 2.40, p = 0.005, ŋ_p_^2^ = 0.67, OP = 0.99). The separate analysis of variance (ANOVAs) results are described below. All mean and standard deviation values, as well as confidence intervals, are presented in the Supporting Information (S1 Table).

#### Time domain

The ANOVA showed a significant effect for Moments on the Mean RR (F_2, 34_ = 23.4, p< 0.001, ŋ_p_^2^ = 0.58, OP = 1.00), Mean HR (F_2, 34_ = 29.2, p< 0.001, ŋ_p_^2^ = 0.63, OP = 1.00), SDNN (F_2, 34_ = 6.33, p = 0.006, ŋ_p_^2^ = 0.27, OP = 0.84), RMSSD (F_2, 34_ = 33.1, p< 0.001, ŋ_p_^2^ = 0.66, OP = 1.00), and pNN50 (F_2, 34_ = 9.61, p< 0.001, ŋ_p_^2^ = 0.66, OP = 1.00) indices. For the comparisons between the sitting versus 45° and sitting versus 90° positions, there was a significant reduction in the Mean RR (p = 0.002; <0.001), SDNN (p = 0.007; 0.011), RMSSD (p< 0.001; <0.001), and pNN50 (p = 0.007; 0.002) indices and a significant increase in Mean HR (p< 0.001; <0.001). When we compared the position at 45° versus 90°, there was a significant reduction in the Mean RR (p = 0.002) and RMSSD (p = 0.013) indices and an increase in Mean HR (p = 0.002).

**Fig 3:**
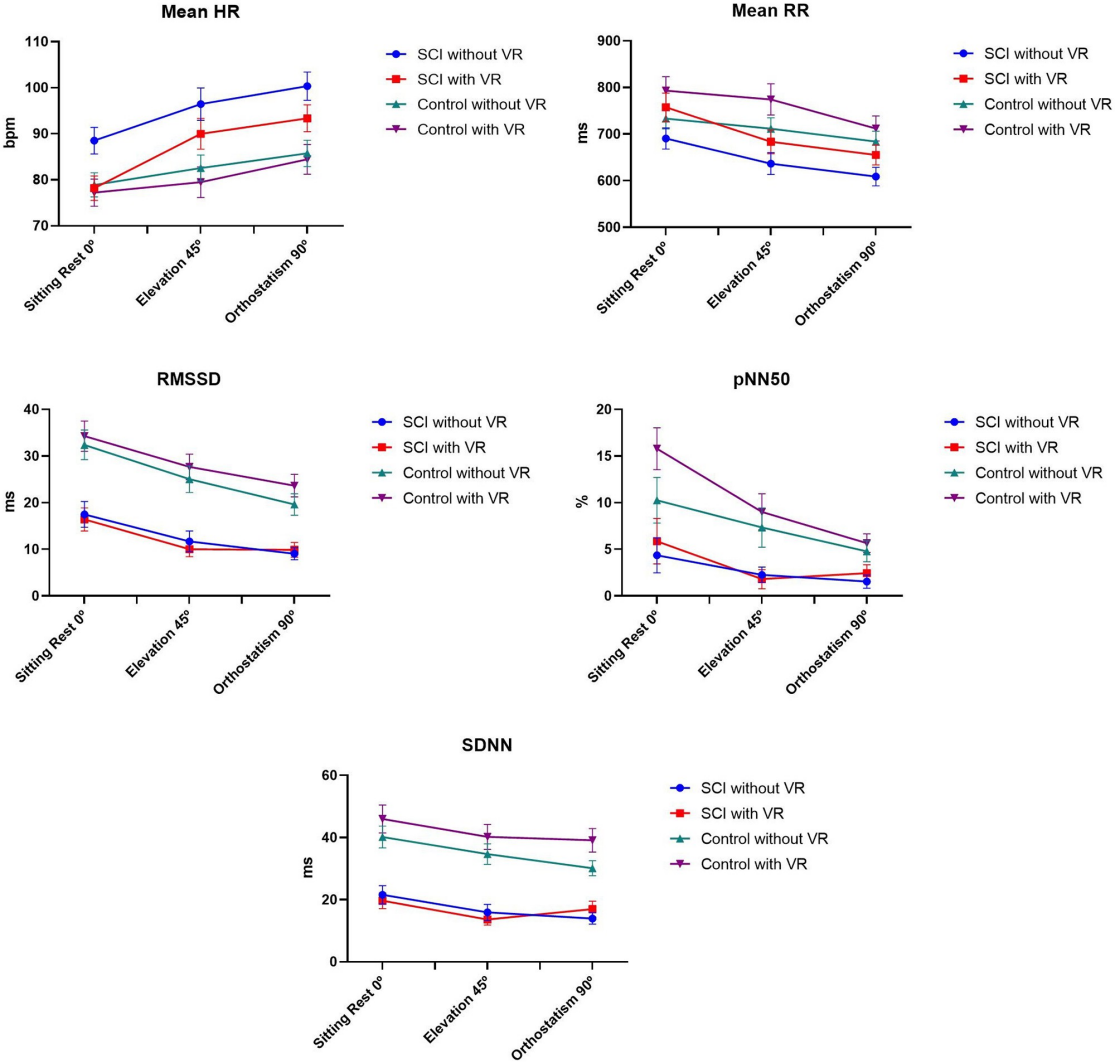
Representation of the mean and standard error of the time domain indices for SCI and able-bodied controls, at the three moments (Sitting rest 0°, elevation 45°, and orthostatism 90°), with and without virtual reality. SCI: spinal cord injury; VR: Virtual Reality activity; bpm: beats per minute; ms: millisecond; HR: heart rate; RR intervals: intervals between heartbeats; RMSSD: square of the root mean difference of successive RR intervals; pNN50: percentage of adjacent RR intervals with a difference in duration greater than 50 milliseconds; SDNN: standard deviation of the mean of all RR intervals over a period.

We found significant main effects for Groups in the SDNN (F_1, 17_ = 7.01, p = 0.017, ŋ_p_^2^ = 0.29, OP = 0.70) index, in which the SCI group showed a lower SDNN than the able-bodied control group (Fig 3). The RMSSD index indicated a trend for a difference between groups that was not proven by statistics. No main effects were observed for VR.

Significant interactions were found of Moments by VR Groups for RMSSD (F_2, 34_ = 3.54, p = 0.046, ŋ_p_^2^ = 0.17, OP = 0.59). The post hoc tests pointed out that only the SCI group that performed the task in VR showed no significant difference between the sitting versus 90° positions in the measured indices, as well as in SDNN.

#### Frequency domain

**Fig 4:**
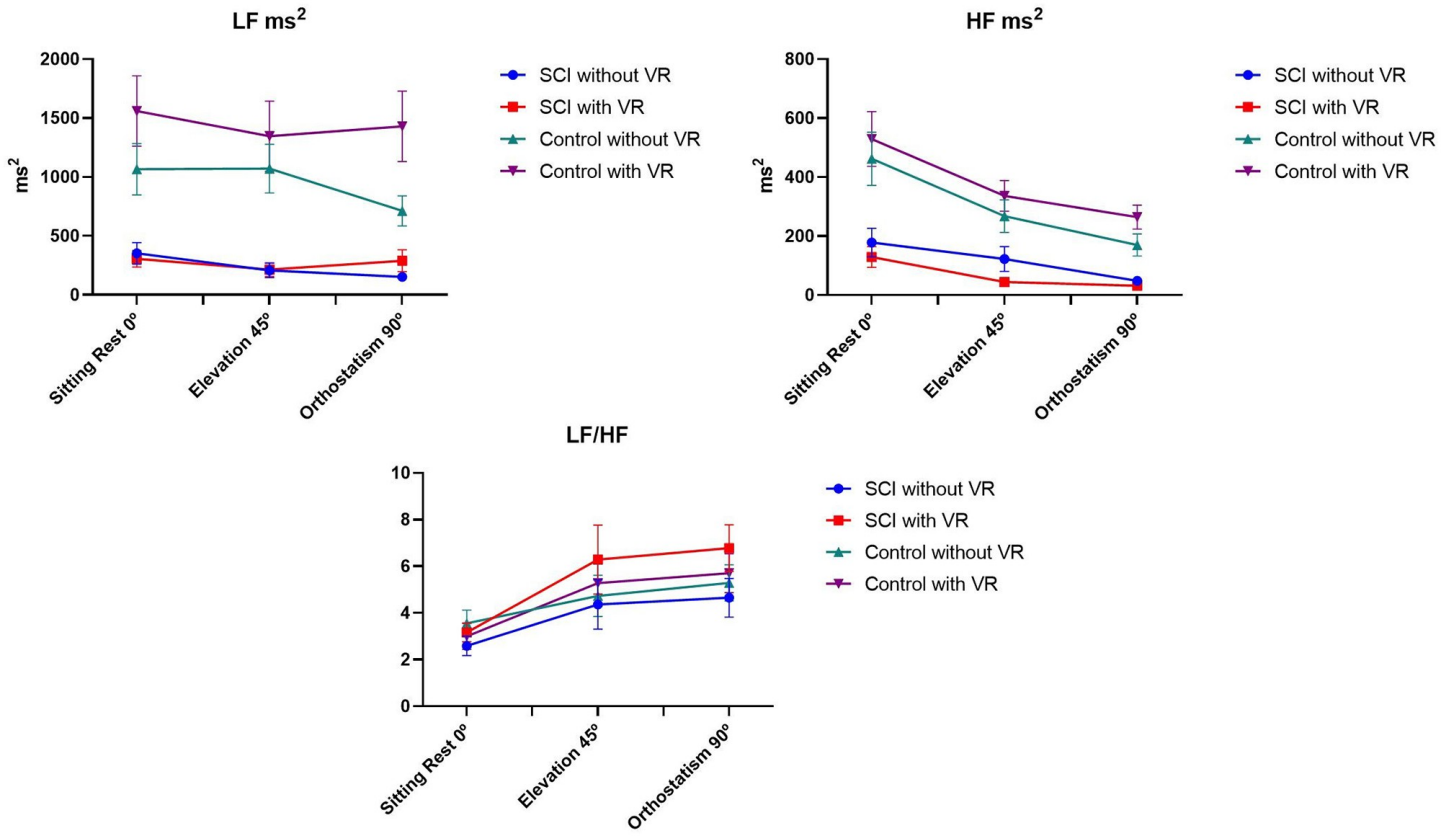
Representation of the mean and standard error of the indices of the frequency domain for SCI and able-bodied controls, at the three moments (Sitting rest 0°, elevation 45°, and orthostatism 90°), with and without virtual reality. SCI: spinal cord injury; VR: Virtual Reality activity; bpm: beats per minute; ms²: millisecond squared; HF: high frequency; LF: low frequency; LF / HF: ratio of low frequency and high frequency.

Regarding the FD indices we found similar results to the TD indices. The main effect for Moments showed a significant difference for the indices HF ms^2^ (F_2, 34_ = 13.0, p< 0.001, ŋ_p_^2^ = 0.43, OP = 0.99) and LF/HF (F_2, 34_ = 7.50, p = 0.004, ŋ_p_^2^ = 0.31, OP = 0.88) and a significant interaction between Moments, Groups, and VR for the LF/HF ratio (F_2, 34_ = 3.88, p = 0.030, ŋ_p_^2^ = 0.19, OP = 0.60). The post hoc test demonstrated that, as in the TD indices, only the SCI group that performed the task in VR did not show a significant difference between the sitting versus 90° positions in the HF ms^2^ index. As with the LF/HF ratio, in both the SCI group and the able-bodied control group that performed the activity in VR there was a significant difference between the sitting versus 90° positions (Fig 4).

There was a main effect for Groups (F_1, 17_ = 7.31, p = 0.015, ŋ_p_^2^ = 0.30, OP = 0.72) in the LF ms^2^ index. This result points to a lower value of LF ms^2^ in the SCI group when compared to the able-bodied control group.

#### Poincaré plot, sympathetic, parasympathetic and stress indices

The main effect for Moments showed a significant difference for SD1 (F_2, 34_ = 33.1, p< 0.001, ŋ_p_^2^ = 0.66, OP = 1.00), SD2 (F_2, 34_ = 4.45, p = 0.023, ŋ_p_^2^ = 0.21, OP = 0.69), SD2/SD1 (F_2, 34_ = 21.2, p< 0.001, ŋ_p_^2^ = 0.56, OP = 1.00), PNSi (F_2, 34_ = 39.5, p< 0.001, ŋ_p_^2^ = 0.70, OP = 1.00), SNSi (F_2, 34_ = 33.6, p< 0.001, ŋ_p_^2^ = 0.66, OP = 1.00), and the Stress index (F_2, 34_ = 9.75, p< 0.001, ŋ_p_^2^ = 0.37, OP = 0.97) and an interaction between Moments, Groups, and VR for the SD1 index (F_2, 34_ = 3.54, p = 0.040, ŋ_p_^2^ = 0.17, OP = 0.59). The post-hoc test showed that for the SD1 and SD2 indices there was a reduction in the values from the sitting at rest position to elevation at 45° (p <0.001 and 0.018) and to orthostatism at 90° (p <0.001 and 0.036), and from 45° to 90° there was a reduction only in the SD1 index. For the SD2/SD1 ratio there was an increase from the sitting at rest position to 90° (p <0.001) and from 45° to 90° (p <0.001).

**Fig 5:**
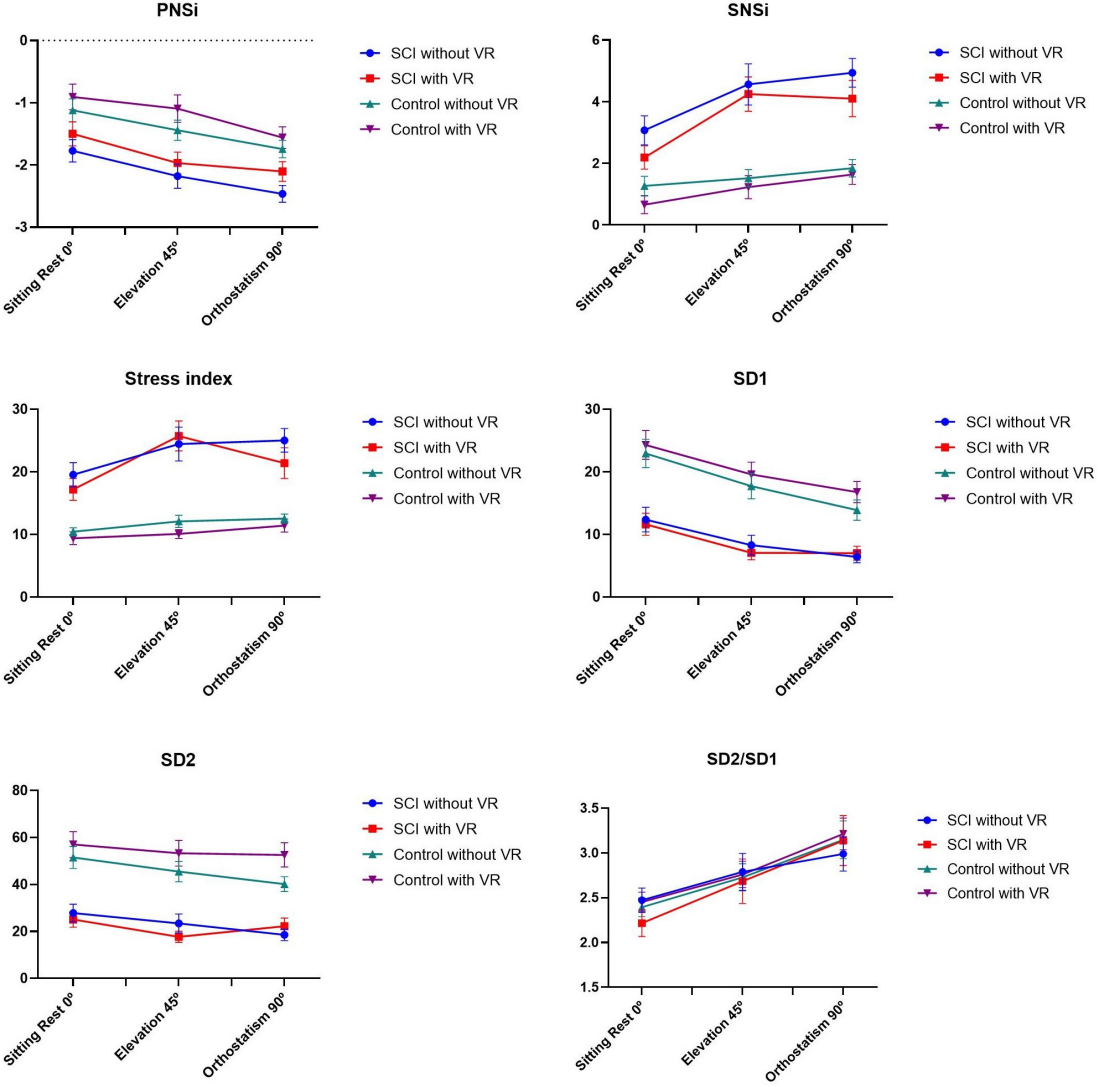
Representation of the mean and standard error of the sympathetic (SNSi), parasympathetic (PNSi), and stress (Stress index) indices for SCI and able-bodied controls, at the three moments (Sitting rest 0°, elevation 45°, and orthostatism 90°), with and without virtual reality. SCI: spinal cord injury; VR: Virtual Reality activity; PNSi: index of parasympathetic nervous system; SNSi: index of sympathetic nervous system; Stress index: geometric measure of HRV that reflects the stress of the cardiovascular system; SD1: the standard deviation of instantaneous beat-to-beat RR interval variability in ms; SD2: standard deviation of continuous long-term RR interval variability in ms; SD2/SD1: ratio of short and long RR interval variations.

The main effect for groups was found only in the SD2 (F_2, 34_ = 7.97, p = 0.012, ŋp2 = 0.32, OP = 0.76) and Stress index (F_2, 34_ = 8.85, p = 0.009, ŋp2 = 0.34, OP = 0.80) in which the SCI group had a lower SD2 and a higher Stress index (Fig 5).

### Correlation

To analyze which of the independent variables (age, ASIA, FIM, SCIM-III, and neurological level) are associated with higher or lower HRV, Pearson’s correlation was performed. The results showed that of these variables, only the neurological level is associated with lower HRV, that is, individuals with higher lesions have lower values of SDNN (*r* = 0.41, p = 0.011), RMSSD (*r* = 0.37, p = 0.024), and SD1 (*r* = 0.37, p = 0.024).

## Discussion

The present study investigated the autonomic modulation of individuals with SCI and able-bodied controls at rest and while performing a motor-cognitive virtual task during postural changes from sitting to orthostatism. The results partially confirmed our hypotheses, postural changes influenced the HRV in individuals with SCI and the use of a motor-cognitive virtual task provided better autonomic adaptation. However, the SCI individuals presented lower HRV compared with the able-bodied individuals in all postures. These results will be discussed below.

### 1- Comparison between spinal cord injury and able-bodied controls

We found that individuals with SCI had lower HRV in three indices (SDNN, RMSSD, and SD1) and a higher Stress index in all postures compared to able bodied controls. According to Baevsky and Berseneva [49], the Stress index usually increases during exercise, and stress symptoms are evoked by the sympathetic system, reflecting high sympathetic cardiac activation and reduced variability. More interestingly, the lower HRV in SCI individuals was related to the level of injury, which may be associated with cardiovascular stress and secondary conditions that characterize SCI. Although Kyriakides et al. [21] reiterate the decrease in cardiac autonomic modulation in all SCI individuals, our results demonstrated that high cervical and thoracic injuries presented lower global variability (SDNN), and lower parasympathetic action (RMSSD and SD1). According to Popa et al. [54], plasma levels of catecholamines, both adrenaline and noradrenaline, are also influenced by the level of injury and respond with changing rates according to orthostatism or the individual’s inclination.

### 2- Postural changes and heart rate variability

Our results showed that with postural change there were modifications in the HRV parameters in both groups (SCI and able-bodied control), with a significant increase in Mean HR, and sympathetic indices (SNSi and Stressi) as the angulation increased, peaking in the orthostatism position, and in parallel, significant reductions in HRV parameters, such as time between heart beats (Mean RR) and parasympathetic activity (RMSSD, PNN50, HF ms^2^, PNSi).

Thus, when the individual assumes the orthostatism position there is a greater accumulation of blood in the lower regions of the body, with a consequent reduction in venous return and a tendency to hypotension, however, the baroreceptors are triggered to cause sympathetic activation and vagal inhibition, justifying the increase in HR in both the SCI and able-bodied control groups [55–57].

Moreover, we found an important increase in the sympathovagal balance during postural transition in both groups (SCI and able-bodied control). In this context the study of Jan et al. [58], described that sympathovagal balance increases in an upright posture and decreases in a sitting posture.

### 3- Virtual reality task in spinal cord injury

Although both groups presented an influence of postural changes and lower HRV, it was more evident in the SCI group. An important result was that only the SCI group that performed the VR task showed no significant difference between the sitting versus orthostatism positions in the parasympathetic system (SDNN, RMSSD, and pNN50 indices). However, this group (SCI that practiced VR task) was the only one that presented a higher increase in the sympathovagal balance (LF/HF ratio) when changing position from sitting to orthostatism (i.e., this change means an increase in sympathetic activity that represents a more normal ANS adaptation). These results are important for rehabilitation considering that SCI individuals present lower sympathetic dominance [16, 18, 21, 58] and a VR task could represent a possibility to improve ANS activity.

Considering the possibility of syncope and loss of consciousness that can occur during postural change in individuals with motor limitations (inadequate elevation of HR and orthostatic intolerance) the rehabilitation professional needs to be prepared, using passive and paused movements, with gradual vertical elevation to keep the blood pressure as stable as possible [59–61]. Thus, we emphasize that our results collaborate with the possibility of using a motor-cognitive task during the position changes, which can provide an increase in the sympathovagal system, with better ANS activation.

We can only speculate that the VR tasks used provide a motor-cognitive stimulus that influences the ANS functioning. Attentional, emotional, and motor factors cooperate in the self-regulation process, so that higher levels of sympathovagal balance may be justified by increased activity in the executive regions of the brain when practicing the VR task [62–64].

Corroborating our findings, Qian et al. [65] state that VR tasks have the potential to exert a positive impact on the physiological, psychological, and rehabilitative outcomes of the individual. Although we found positive results with the use of a motor-cognitive VR task during postural change, we agree with the review of Araújo et al. [33] which indicated that the use of VR in the rehabilitation of individuals with SCI should be further explored, as it opens the possibility for a variety of tasks and possibilities that can be implemented, as well as presenting motivation and engagement [66, 67].

### 4—Limitations and future studies

Although we found interesting results, we can point out some limitations of the present study: (1) the participants were evaluated at a single time point (cross-sectional study) and a longitudinal protocol could provide deeper information on the effects of a motor-cognitive task in ANS adaptation; (2) we did not assess blood pressure, respiratory rate, and rating of perceived exertion during the protocol (these data should be investigated in future studies); (3) we used a laboratory specific motor-cognitive task and other VR tasks and exergames should be assessed in future to analyze engagement, motivation, and influence on HRV.

## Conclusion

Individuals with spinal cord injury presented lower heart rate variability indices when compared with able-bodied controls and individuals with higher SCI lesions presented lower values of global variability and parasympathetic activity. More importantly, the use of VR tasks seems to contribute to better sympathovagal balance during postural changes and this new technology could represent a future strategy for use in physical rehabilitation of SCI individuals.

## Supporting information

S1 Checklist Inclusivity in global research. Ethical considerations, permits, authorship and Human subject’s research.
(PDF)

S1 Table Continuous data. Presentation of measures of central tendency and dispersion of HRV indices for Moments, Groups, and Virtual Reality.
(PDF)
