# Supplementary material for: Cardiac autonomic modulation in response to postural transition during a virtual reality task in individuals with spinal cord injury: A cross-sectional study
Source: PLoS One. 2023 Apr 13;18(4):e0283820. doi: 10.1371/journal.pone.0283820 (PMC10101494; doi:10.1371/journal.pone.0283820)
Supplement: S1 Table — Presentation of measures of central tendency and dispersion of HRV indices for Moments, Groups, and Virtual Reality. (PDF) [file pone.0283820.s002.pdf]

S1 Table. Supporting Information Table. Continuous data. Presentation of measures of central tendency and dispersion of HRV indices for Moments, Groups, and Virtual Reality.

Variables	Moments	Groups			
		**SCI without VR** Mean (SD) [95% CI: LB; UB]	**SCI with VR** Mean (SD) [95% CI: LB; UB]	**Control without VR** Mean (SD) [95% CI: LB; UB]	**Control with VR** Mean (SD) [95% CI: LB; UB]
**Mean Heart Rate (bpm)**	Rest	88.5 (11.9) [83.6; 93.7]	77.5 (10.5) [73.4; 81.8]	78.9 (10.5) [74.6; 83.6]	77.8 (11.2) [72.8; 82.7]
	45º	96.0 (14.6) [89.9; 102.3]	89.9 (14.3) [84.5; 96.0]	82.4 (11.3) [77.4; 87.1]	78.8 (12.7) [73.0; 84.7]
	90 º	100.3 (13.4) [94.6; 105.8]	93.3 (12.7) [88.5; 98.2]	85.4 (10.9) [81.0; 90.2]	84.4 (12.9) [78.3; 90.4]
**Mean RR intervals (ms)**	Rest	691.1 (93.5) [650.9; 729.2]	760.8 (133.8) [705.7; 811.1]	736.1 (84.1) [699.9; 768.8]	787.1 (115.0) [736.8; 839.2]
	45º	638.7 (97.8) [597.9; 679.6]	684.9 (108.8) [642.3; 728.5]	714.6 (94.3) [674.7; 751.6]	781.4 (128.1) [722.0; 841.0]
	90 º	608.7 (86.1) [574.4; 645.8]	655.4 (93.0) [620.3; 692.1]	688.6 (86.2) [652.6; 722.8]	712.5 (100.6) [666.7; 760.5]
**SDNN (ms)**	Rest	21.6 (12.2) [16.8; 27.1]	20.6 (11.0) [16.3; 25.1]	40.2 (14.8) [33.8; 47.2]	44.2 (16.7) [36.3; 52.4]
	45º	15.3 (10.2) [11.1; 20.0]	13.8 (7.7) [10.7; 16.8]	35.4 (14.3) [29.3; 41.7]	41.3 (14.8) [34.6; 48.4]

	90 º	13.9 (7.9)<br>[10.7; 17.8]	17.3 (11.3)<br>[12.8; 22.1]	30.7 (10.0)<br>[26.5; 35.0]	38.6 (14.3)<br>[32.1; 45.2]
**RMSSD (ms)**	Rest	17.5 (11.4)<br>[12.8; 22.5]	17.2 (10.6)<br>[13.0; 21.8]	32.3 (13.5)<br>[26.7; 38.0]	33.2 (11.7)<br>[27.6; 39.2]
	45º	11.3 (8.7)<br>[7.9; 15.6]	10.1 (6.6)<br>[7.6; 13.0]	25.3 (12.2)<br>[20.5; 30.6]	28.7 (10.2)<br>[23.9; 33.5]
	90 º	9.0 (5.7)<br>[6.9; 11.9]	10.1 (6.7)<br>[7.6; 12.9]	19.8 (9.4)<br>[16.1; 24.1]	23.2 (9.2)<br>[18.6; 27.6]
**pNN50 (%)**	Rest	5.7 (6.2)<br>[3.2; 8.5]	6.4 (7.1)<br>[3.8; 9.6]	10.3 (9.8)<br>[6.2; 14.8]	16.4 (7.7)<br>[12.6; 20.2]
	45º	2.1 (1.7)<br>[1.4; 2.9]	2.1 (2.2)<br>[1.3; 3.0]	6.6 (7.3)<br>[3.9; 10.1]	9.7 (7.5)<br>[6.4; 13.5]
	90 º	1.9 (1.7)<br>[1.1; 2.7]	3.1 (2.1)<br>[2.2; 4.0]	4.7 (3.7)<br>[3.3; 6.4]	5.7 (3.3)<br>[4.2; 7.3]
**LF (ms^2)**	Rest	358.4 (372.7)<br>[214.6; 534.1]	322.8 (296.8)<br>[206.0; 455.6]	1090.6 (890.2)<br>[755.2; 1547.2]	1519.4 (1078.3)<br>[1038.7; 2033.2]
	45º	194.1 (245.3)<br>[101.8; 315.8]	206.7 (252.5)<br>[116.3; 317.3]	1111.5 (894.2)<br>[750.3; 1514.0]	1752.5 (1320.5)<br>[1169.5; 2402.7]
	90 º	152.3 (163.4)<br>[87.6; 236.6]	311.3 (415.9)<br>[168.7; 493.7]	753.1 (559.1)<br>[539.1; 1014.1]	1411.5 (1119.1)<br>[927.4; 1935.1]
**HF (ms^2)**	Rest	181.8 (199.6)<br>[105.3; 273.1]	138.4 (149.5)<br>[81.1; 203.1]	448.4 (388.2)<br>[292.6; 620.5]	547.9 (328.1)<br>[391.5; 708.1]

	45º	116.6 (175.4)	51.7 (73.9)	273.4 (225.3)	343.4 (196.9)
		[47.4; 196.2]	[24.6; 84.5]	[177.1; 368.1]	[245.4; 440.9]
	90 º	48.7 (53.1)	40.7 (54.5)	165.3 (150.5)	256.6 (155.0)
		[27.5; 74.7]	[21.2; 66.4]	[107.5; 233.4]	[181.5; 329.0]
**LF/HF**	Rest	2.6 (1.6)	3.2 (1.8)	3.5 (2.4)	2.8 (2.0)
		[2.0; 3.3]	[2.4; 4.0]	[2.5; 4.6]	[2.0; 3.9]
	45º	4.1 (4.2)	6.7 (6.1)	4.7 (3.3)	5.5 (3.8)
		[2.4; 6.0]	[4.4; 9.2]	[3.4; 6.4]	[3.9; 7.5]
	90 º	4.7 (3.6)	6.6 (4.2)	5.1 (2.9)	5.7 (3.3)
		[3.2; 6.3]	[5.0; 8.3]	[3.9; 6.3]	[4.1; 7.3]
**SD1 (ms)**	Rest	12.4 (8.1)	12.2 (7.5)	22.8 (9.6)	24.8 (8.2)
		[9.1; 15.9]	[9.2; 15.5]	[18.9; 26.9]	[20.8; 28.5]
	45º	8.0 (6.2)	7.5 (5.3)	17.9 (8.6)	20.8 (7.5)
		[5.6; 11.0]	[5.5; 9.9]	[14.5; 21.7]	[17.1; 24.3]
	90 º	6.4 (4.0)	7.2 (4.7)	14.0 (6.6)	16.4 (6.5)
		[4.9; 8.4]	[5.4; 9.2]	[11.4; 17.1]	[13.1; 19.5]
**SD2 (ms)**	Rest	27.9 (15.4)	26.3 (14.0)	51.7 (19.9)	57.4 (19.4)
		[21.9; 34.8]	[20.9; 32.1]	[43.3; 61.0]	[48.0; 65.7]
	45º	22.6 (17.1)	18.7 (11.6)	46.5 (18.9)	56.9 (21.4)
		[15.7; 30.4]	[14.2; 23.5]	[38.4; 54.9]	[47.3; 67.2]
	90 º	18.5 (10.6)	22.7 (15.0)	40.9 (13.0)	52.0 (19.5)
		[14.3; 23.8]	[16.9; 29.1]	[35.4; 46.4]	[43.1; 61.0]
**SD2/SD1**	Rest	2.5 (0.6)	2.2 (0.6)	2.4 (0.4)	2.5 (0.4)
		[2.2; 2.7]	[2.0; 2.5]	[2.2; 2.6]	[2.3; 2.7]

	45º	2.7 (0.9) [2.4; 3.2]	2.7 (1.0) [2.3; 3.1]	2.8 (0.6) [2.5; 3.0]	2.7 (0.5) [2.5; 3.0]
	90 º	3.0 (0.8) [2.6; 3.3]	3.1 (1.3) [2.7; 3.6]	3.1 (0.8) [2.8; 3.5]	3.2 (0.7) [2.9; 3.5]
**PNS index**	Rest	-1.8 (0.7) [-2.1; -1.5]	-1.5 (0.8) [-1.9; -1.2]	-1.1 (0.7) [-1.4; -0.8]	-1.0 (0.7) [-1.3; -0.6]
	45º	-2.2 (0.8) [-2.5; -1.8]	-2.0 (0.8) [-2.2; -1.6]	-1.4 (0.6) [-1.7; -1.2]	-1.0 (0.8) [-1.4; -0.6]
	90 º	-2.5 (0.6) [-2.7; -2.2]	-2.1 (0.7) [-2.4; -1.8]	-1.7 (0.5) [-1.9; -1.5]	-1.6 (0.7) [-1.9; -1.3]
**SNS index**	Rest	3.1 (1.8) [2.3; 3.9]	2.0 (1.5) [1.4; 2.7]	1.3 (1.3) [0.7; 1.8]	0.7 (1.1) [0.2; 1.1]
	45º	4.6 (2.8) [3.4; 5.7]	4.2 (2.4) [3.3; 5.2]	1.5 (1.1) [1.0; 2.0]	1.2 (1.4) [0.5; 1.9]
	90 º	5.0 (2.0) [4.2; 5.8]	4.0 (2.6) [3.0; 5.2]	1.8 (1.0) [1.4; 2.2]	1.6 (1.3) [1.1; 2.2]
**Stress index**	Rest	19.6 (7.5) [16.4; 22.7]	16.6 (6.7) [14.0; 19.5]	10.4 (2.5) [9.3; 11.5]	9.8 (3.7) [8.3; 11.7]
	45º	24.8 (11.2) [20.2; 29.6]	25.4 (10.3) [21.3; 29.8]	12.0 (3.9) [10.5; 13.7]	10.0 (2.6) [8.7; 11.2]
	90 º	25.3 (7.9) [21.9; 28.8]	20.4 (10.6) [16.2; 24.8]	12.4 (2.6) [11.4; 13.6]	11.4 (4.2) [9.6; 13.4]

HRV: heart rate variability; SCI: spinal cord injury; VR: Virtual Reality activity; SD: standard deviation; CI: confidence interval presented in Lower Limit (LB) and Upper Limit (UB); bpm: beats per minute; HF: high frequency; LF: low frequency;

LF / HF: ratio of low frequency and high frequency; ms: millisecond; ms^2: millisecond squared; RMSSD: square of the root mean difference of successive RR intervals; RR intervals: intervals between heartbeats; SDNN: standard deviation of the mean of all RR intervals over a period; pNN50: percentage of adjacent RR intervals with a difference in duration greater than 50 milliseconds SD1: the standard deviation of instantaneous beat-to-beat RR interval variability in ms; SD2: standard deviation of continuous long-term RR interval variability in ms; SD1 / SD2: ratio of short and long RR interval variations; PNS: parasympathetic nervous system; SNS: sympathetic nervous system.
